# Supplementary material for: Quantifying the effect of isolation and negative certification on COVID-19 transmission
Source: Sci Rep. 2023 Jul 12;13:11264. doi: 10.1038/s41598-023-37872-w (PMC10338484; doi:10.1038/s41598-023-37872-w)
Supplement: Supplementary file 1 — Supplementary Information. [file 41598_2023_37872_MOESM1_ESM.pdf]

Supplementary information

Derivation of the conditional probability of non-infection status given one negative PCR test result

This clause derives the non-infected probability when an individual has a negative test result on day 0. In other words, $\mathscr{P}(S_0 \cup R_0 | \ominus_0)$ is derived. Note that cursive script $\mathscr{P}$ stands for the probability, while capital letter P stands for an individual's status. Let $S'_0 := S_0 \cup R_0$; that is, S'_0 means a non-infection status on day 0. Since the overall status on day 0, say Ω_0, equals $S_0 \cup E_0 \cup P_{1,0} \cup P_{2,0} \cup I_0 \cup R_0$, the following holds from Bayes' theorem.

$$
\begin{aligned}
&\mathscr{P}(S_0 \cup R_0 | \ominus_0) \\
&= \frac{\mathscr{P}(S'_0) P(\ominus_0 | S'_0)}{\mathscr{P}(S'_0)\mathscr{P}(\ominus_0|S'_0) + \mathscr{P}(E_0)\mathscr{P}(\ominus_0|E_0) + \mathscr{P}(P_{1,0})\mathscr{P}(\ominus_0|P_{1,0})\mathscr{P}(P_{2,0})\mathscr{P}(\ominus_0|P_{2,0}) + \mathscr{P}(I_0)\mathscr{P}(\ominus_0|I_0)} \\
&= \frac{b\mathscr{P}(S'_0)}{b\mathscr{P}(S'_0) + b\mathscr{P}(E_0) + (1-a_1)\mathscr{P}(P_{1,0}) + (1-a_2)\mathscr{P}(P_{2,0}) + (1-a_s)\mathscr{P}(I_0)}.
\end{aligned}
$$

Derivation of the conditional probability of non-infected status given two negative PCR test results

This clause derives the non-infection probability when an individual has a negative PCR test result on both day 0 and day k k $(1 \leq k \leq 14)$. In other words, $\mathscr{P}(S_k \cup R_k | \ominus_0 \cap \ominus_k)$ is derived. Let $S'_k := S_k \cup R_k$; that is, S'_k indicates non-infection status at day k $(0 \leq k \leq 14)$. First, from Bayes' theorem, the following holds.

$$
\begin{aligned}
&\mathscr{P}\left(S_k \cup R_k \middle| \ominus_0 \cap \ominus_k\right) \\
&\quad = \mathscr{P}(S'_k)\mathscr{P}(\ominus_0 \cap \ominus_k | S'_k) \Big/ \\
&\quad \Big(\mathscr{P}(S'_k)\mathscr{P}(\ominus_0 \cap \ominus_k|S'_k) + \mathscr{P}(E_k)\mathscr{P}(\ominus_0 \cap \ominus_k|E_k) + \mathscr{P}(P_{1,k})\mathscr{P}(\ominus_0 \cap \ominus_k|P_{1,k}) \\
&\qquad + \mathscr{P}(P_{2,k})\mathscr{P}(\ominus_0 \cap \ominus_k|P_{2,k}) + \mathscr{P}(I_k)\mathscr{P}(\ominus_0 \cap \ominus_k|I_k)\Big). \qquad \text{(S.1)}
\end{aligned}
$$

Next, in order to calculate (S.1), $\mathscr{P}(S'_k)\mathscr{P}(\ominus_0 \cap \ominus_k|S'_k)$ is derived. By the definitions of the conditional probability and Ω_0,

$$
\mathscr{P}(S'_k)\mathscr{P}(\ominus_0 \cap \ominus_k|S'_k) = \mathscr{P}(\ominus_0 \cap \ominus_k \cap S'_k \cap \Omega_0). \qquad \text{(S.2)}
$$

Then, from De Morgan's laws, the following holds.

$$
\begin{aligned}
\text{(S.2)} &= \mathscr{P}\left(\ominus_0 \cap \ominus_k \cap S'_k \cap (S'_0 \cup E_0 \cup P_{1,0} \cup P_{2,0} \cup I_0)\right) \\
&= \mathscr{P}\Big((\ominus_0 \cap \ominus_k \cap S'_k \cap S'_0) \cup (\ominus_0 \cap \ominus_k \cap S'_k \cap E_0) \cup (\ominus_0 \cap \ominus_k \cap S'_k \cap P_{1,0}) \\
&\qquad \cup (\ominus_0 \cap \ominus_k \cap S'_k \cap P_{2,0}) \cup (\ominus_0 \cap \ominus_k \cap S'_k \cap I_0)\Big) \\
&= \mathscr{P}(\ominus_0 \cap \ominus_k \cap S'_k \cap S'_0) + \mathscr{P}(\ominus_0 \cap \ominus_k \cap S'_k \cap E_0) + \mathscr{P}(\ominus_0 \cap \ominus_k \cap S'_k \cap P_{1,0}) \\
&\qquad + \mathscr{P}(\ominus_0 \cap \ominus_k \cap S'_k \cap P_{2,0}) + \mathscr{P}(\ominus_0 \cap \ominus_k \cap S'_k \cap I_0) \\
&= \mathscr{P}(\ominus_0 \cap \ominus_k|S'_k \cap S'_0)\mathscr{P}(S'_k \cap S'_0) + \mathscr{P}(\ominus_0 \cap \ominus_k|S'_k \cap E_0)\mathscr{P}(S'_k \cap E_0) + \mathscr{P}(\ominus_0 \cap \ominus_k|S'_k \cap P_{1,0})\mathscr{P}(S'_k \cap P_{1,0}) \\
&\qquad + \mathscr{P}(\ominus_0 \cap \ominus_k|S'_k \cap P_{2,0})\mathscr{P}(S'_k \cap P_{2,0}) + \mathscr{P}(\ominus_0 \cap \ominus_k|S'_k \cap I_0)\mathscr{P}(S'_k \cap I_0) \\
&= \mathscr{P}(\ominus_0 \cap \ominus_k|S'_k \cap S'_0)\mathscr{P}(S'_0)\mathscr{P}(S'_k|S'_0) + \mathscr{P}(\ominus_0 \cap \ominus_k|S'_k \cap E_0)\mathscr{P}(E_0)\mathscr{P}(S'_k|E_0) \\
&\qquad + \mathscr{P}(\ominus_0 \cap \ominus_k|S'_k \cap P_{1,0})\mathscr{P}(P_{1,0})\mathscr{P}(S'_k|P_{1,0}) + \mathscr{P}(\ominus_0 \cap \ominus_k|S'_k \cap P_{2,0})\mathscr{P}(P_{2,0})\mathscr{P}(S'_k|P_{2,0}) \\
&\qquad + \mathscr{P}(\ominus_0 \cap \ominus_k|S'_k \cap I_0)\mathscr{P}(I_0)\mathscr{P}(S'_k|I_0). \qquad \text{(S.3)}
\end{aligned}
$$

In the same way, the following hold.

$$\begin{aligned}
\mathscr{P}(E_k)\mathscr{P}(\ominus_0\cap\ominus_k|E_k) &= \mathscr{P}\left(\ominus_0\cap\ominus_k|E_k\cap S_0'\right)\mathscr{P}(S_0')\mathscr{P}(E_k|S_0')\\
&+\mathscr{P}\left(\ominus_0\cap\ominus_k|E_k\cap E_0\right)\mathscr{P}(E_0)\mathscr{P}(E_k|E_0)\\
&+\mathscr{P}\left(\ominus_0\cap\ominus_k|E_k\cap P_{1,0}\right)\mathscr{P}(P_{1,0})\mathscr{P}(E_k|P_{1,0})\\
&+\mathscr{P}\left(\ominus_0\cap\ominus_k|E_k\cap P_{2,0}\right)\mathscr{P}(P_{2,0})\mathscr{P}(E_k|P_{2,0})\\
&+\mathscr{P}\left(\ominus_0\cap\ominus_k|E_k\cap I_0\right)\mathscr{P}(I_0)\mathscr{P}(E_k|I_0) \qquad \text{(S.4)}\\
\mathscr{P}(P_{1,k})\mathscr{P}(\ominus_0\cap\ominus_k|P_{1,k}) &= \mathscr{P}\left(\ominus_0\cap\ominus_k|P_{1,k}\cap S_0'\right)\mathscr{P}(S_0')\mathscr{P}(P_{1,k}|S_0')\\
&+\mathscr{P}\left(\ominus_0\cap\ominus_k|P_{1,k}\cap E_0\right)\mathscr{P}(E_0)\mathscr{P}(P_{1,k}|E_0)\\
&+\mathscr{P}\left(\ominus_0\cap\ominus_k|P_{1,k}\cap P_{1,0}\right)\mathscr{P}(P_{1,0})\mathscr{P}(P_{1,k}|P_{1,0})\\
&+\mathscr{P}\left(\ominus_0\cap\ominus_k|P_{1,k}\cap P_{2,0}\right)\mathscr{P}(P_{2,0})\mathscr{P}(P_{1,k}|P_{2,0})\\
&+\mathscr{P}\left(\ominus_0\cap\ominus_k|P_{1,k}\cap I_0\right)\mathscr{P}(I_0)\mathscr{P}(P_{1,k}|I_0) \qquad \text{(S.5)}\\
\mathscr{P}(P_{2,k})\mathscr{P}(\ominus_0\cap\ominus_k|P_{2,k}) &= \mathscr{P}\left(\ominus_0\cap\ominus_k|P_{2,k}\cap S_0'\right)\mathscr{P}(S_0')\mathscr{P}(P_{2,k}|S_0')\\
&+\mathscr{P}\left(\ominus_0\cap\ominus_k|P_{2,k}\cap E_0\right)\mathscr{P}(E_0)\mathscr{P}(P_{2,k}|E_0)\\
&+\mathscr{P}\left(\ominus_0\cap\ominus_k|P_{2,k}\cap P_{1,0}\right)\mathscr{P}(P_{1,0})\mathscr{P}(P_{2,k}|P_{1,0})\\
&+\mathscr{P}\left(\ominus_0\cap\ominus_k|P_{2,k}\cap P_{2,0}\right)\mathscr{P}(P_{2,0})\mathscr{P}(P_{2,k}|P_{2,0})\\
&+\mathscr{P}\left(\ominus_0\cap\ominus_k|P_{2,k}\cap I_0\right)\mathscr{P}(I_0)\mathscr{P}(P_{2,k}|I_0) \qquad \text{(S.6)}\\
\mathscr{P}(I_k)\mathscr{P}(\ominus_0\cap\ominus_k|I_k) &= \mathscr{P}\left(\ominus_0\cap\ominus_k|I_k\cap S_0'\right)\mathscr{P}(S_0')\mathscr{P}(I_k|S_0')\\
&+\mathscr{P}\left(\ominus_0\cap\ominus_k|I_k\cap E_0\right)\mathscr{P}(E_0)\mathscr{P}(I_k|E_0)\\
&+\mathscr{P}\left(\ominus_0\cap\ominus_k|I_k\cap P_{1,0}\right)\mathscr{P}(P_{1,0})\mathscr{P}(I_k|P_{1,0})\\
&+\mathscr{P}\left(\ominus_0\cap\ominus_k|I_k\cap P_{2,0}\right)\mathscr{P}(P_{2,0})\mathscr{P}(I_k|P_{2,0})\\
&+\mathscr{P}\left(\ominus_0\cap\ominus_k|I_k\cap I_0\right)\mathscr{P}(I_0)\mathscr{P}(I_k|I_0). \qquad \text{(S.7)}
\end{aligned}$$

Finally, by substituting (S.3)–(S.7) into (S.1), the desired probability can be derived.

Derivation of the non-infection probability under a negative PCR test result on both day 0 and day 1
From Table 1, the following relationships between the day 0 and day 1 statuses hold.

$$\mathscr{P}(S_1\cup R_1)=\mathscr{P}(S_0\cup R_0)+\frac{1}{7}\mathscr{P}(I_0), \qquad \mathscr{P}(E_1)=\frac{2}{3}\mathscr{P}(E_0),$$
$$\mathscr{P}(P_{1,1})=\frac{1}{3}\mathscr{P}(E_0), \qquad \mathscr{P}(P_{2,1})=\mathscr{P}(P_{1,0}), \quad \text{and} \quad \mathscr{P}(I_1)=\mathscr{P}(P_{2,0})+\frac{6}{7}\mathscr{P}(I_0).$$

Therefore, the members on the right-hand side of (S.1) can be calculated as follows.

$$\begin{aligned}
\mathscr{P}(S_1')\mathscr{P}(\ominus_0\cap\ominus_1|S_1') &= \mathscr{P}\left(\ominus_0\cap\ominus_1|S_1'\cap S_0'\right)\mathscr{P}(S_0')\mathscr{P}(S_1'|S_0')+\mathscr{P}\left(\ominus_0\cap\ominus_1|S_1'\cap I_0\right)\mathscr{P}(I_0)\mathscr{P}(S_1'|I_0)\\
&= b^2\mathscr{P}(S_0')+\frac{1}{7}b(1-a_I)\mathscr{P}(I_0) \qquad \text{(S.8)}\\
\mathscr{P}(E_1)\mathscr{P}(\ominus_0\cap\ominus_1|E_1) &= \mathscr{P}\left(\ominus_0\cap\ominus_1|E_1\cap E_0\right)\mathscr{P}(E_0)\mathscr{P}(E_1|E_0)\\
&= \frac{2}{3}b^2\mathscr{P}(E_0) \qquad \text{(S.9)}\\
\mathscr{P}(P_{1,1})\mathscr{P}(\ominus_0\cap\ominus_1|P_{1,1}) &= \mathscr{P}\left(\ominus_0\cap\ominus_1|P_{1,1}\cap E_0\right)\mathscr{P}(E_0)\mathscr{P}(P_{1,1}|E_0)\\
&= \frac{1}{3}b(1-a_1)\mathscr{P}(E_0) \qquad \text{(S.10)}\\
\mathscr{P}(P_{2,1})\mathscr{P}(\ominus_0\cap\ominus_1|P_{2,1}) &= \mathscr{P}\left(\ominus_0\cap\ominus_1|P_{2,1}\cap P_{1,0}\right)\mathscr{P}(P_{1,0})\mathscr{P}(P_{2,1}|P_{1,0})\\
&= (1-a_1)(1-a_2)\mathscr{P}(I_{1,0}) \qquad \text{(S.11)}\\
\mathscr{P}(I_1)\mathscr{P}(\ominus_0\cap\ominus_1|I_1) &= \mathscr{P}\left(\ominus_0\cap\ominus_1|I_1\cap P_{2,0}\right)\mathscr{P}(P_{2,0})\mathscr{P}(I_1|P_{2,0})+\mathscr{P}\left(\ominus_0\cap\ominus_1|I_1\cap I_0\right)\mathscr{P}(I_0)\mathscr{P}(I_1|I_0)\\
&= (1-a_2)(1-a_I)\mathscr{P}(P_{2,0})+\frac{6}{7}(1-a_I)^2\mathscr{P}(I_0). \qquad \text{(S.12)}
\end{aligned}$$

By substituting (S.8)–(S.12) and $k = 1$ into (S.1), the desired probability can be derived as follows.

$$
\begin{aligned}
&\mathscr{P}\left(S_1 \cup R_1 \,\middle|\, \ominus_0 \cap \ominus_1\right) \\
&= \left(b^2 \mathscr{P}(S_0') + \frac{1}{7} b(1-a_I)\mathscr{P}(I_0)\right) \Big/ \\
&\quad \left(b^2 \mathscr{P}(S_0') + \frac{1}{7} b(1-a_I)\mathscr{P}(I_0) + \frac{2}{3} b^2 \mathscr{P}(E_0) + \frac{1}{3} b(1-a_1)\mathscr{P}(E_0) + (1-a_1)(1-a_2)\mathscr{P}(P_{1,0}) \right. \\
&\qquad \left. + (1-a_2)(1-a_I)\mathscr{P}(P_{2,0}) + \frac{6}{7}(1-a_I)^2 \mathscr{P}(I_0)\right).
\end{aligned}
$$
